# Psychotherapy in Jordan: An Investigation of the Host and Syrian Refugee Community’s Perspectives

**DOI:** 10.3389/fpsyt.2019.00556

**Published:** 2019-08-13

**Authors:** Carine Karnouk, Kerem Böge, Eric Hahn, Judith Strasser, Stephanie Schweininger, Malek Bajbouj

**Affiliations:** Department of Psychiatry and Psychotherapy, Charité-Universitätsmedizin, Berlin, Germany

**Keywords:** mental health, psychotherapy, Arab, Jordan, refugees, stigma, bias, satisfaction

## Abstract

Little is known about patient satisfaction, bias, stigma, and the effects of psychotherapy within the Kingdom of Jordan or the Arab world in general. The purpose of this study was to explore the perceptions of both the Jordanian host and refugee community members from the psychotherapeutic services offered at various mental health care settings in Jordan. A sample of 100 patients who received psychosocial expert interventions was recruited between October and December 2017 in Amman, Jordan. Participants were either from the host or Syrian refugee community or contacted through multiple organizations working in the mental health field. The Patient Satisfaction Questionnaire, which consists of four subscales covering 1) patient satisfaction, 2) bias toward therapy, 3) effects of therapy, and 4) stigma, was administered. As a means of investigation and exploration, descriptive statistics of participant responses are displayed. Results revealed overall high rates of satisfaction with provided services and perceived benefits of psychotherapeutic interventions. However, respondents showed ambivalence regarding bias and stigma. Subsample analyses indicated no significant differences between both communities. These findings give an understanding of perceptions surrounding psychotherapy in Jordan and some insights on therapeutic processes that may be useful for clinical applications and future research.

## Introduction

In the last decade, political turmoil and unstable social climates have spread across many countries in the Arab region (1–3) . The effect of these volatile circumstances and related stressful experiences has left many individuals at risk of developing a mental illness, with little to no access to mental health care (3). Despite growing efforts in the development of mental health care structures and treatment providers to serve these vulnerable populations, the treatment gap remains high (2–6).

In that context, the Kingdom of Jordan is a relatively small, middle-income country located in the Middle East with a population of nearly 10 million inhabitants (7, 8). Similar to other Arab countries, the health care infrastructure in Jordan is highly urbanized (5), and the country’s social climate is heavily influenced by ongoing political conflicts in nearby countries, which has led to an increase in poverty rates and the resettlement of refugee populations within the country (9, 10). According to a UN report, about 14.4% of the population live under the internationally specified poverty line (8). Furthermore, it has been indicated that only an estimated 305 individuals per 100,000 inhabitants are diagnosed with mental illness (2), indicating an existing diagnostic and treatment gap. As a consequence, rates of psychological distress (39%) and prevalence of mental disorders (26.3%) are high ranking (11). Worldwide, the prevalence of psychological distress ranges roughly between 5% and 27%. It has been reported to be even higher in certain segments of the population that face stressful situations, such as economic, work, or immigration-related conditions (12).

High prevalence rates contrast with only a small number of mental health institutions. In the whole of Jordan, there are only three mental health hospitals for adults, one specialized psychiatric hospital for children (8, 13), and only 64 outpatient facilities (2). In total, there are only two psychiatrists, 0.27 psychologists and 0.04 nurses for every 100,000 inhabitants in Jordan (8, 13)—a pattern that is present and known throughout the whole Middle East (14–16).

These numbers give insight into the current state of affairs regarding mental health care in Jordan, where the population size has significantly grown between the years 2004 and 2015 (7, 8). In addition, ongoing conflicts in Syria have led Jordan to host around 660,000 Syrian refugees, and the numbers are still rising (8). This high number of displaced individuals in need of humanitarian assistance and psychiatric care contributes to an even larger treatment gap. Evidently, the arrival of the Syrian refugee population, with a wide range of affective disorders, is causing additional challenges to Jordan’s already strained mental health care system (8). Some reports even state treatment gaps as high as a 94% in Jordan if all severity levels of mental illnesses are taken into consideration (6). These numbers are similar to those observed in other developing countries ranging between 76.3% and 85.4% (16).

A lack of psychiatric facilities, culturally sensitive treatment programs, and professional training and poor coordination between clinicians and centralized treatment services in metropolitan cities (2, 5), coupled with further social barriers, have been identified as major challenges in the access and delivery of mental health services (5, 10, 17, 18). Some of these barriers include bias toward therapy, beliefs about mental illness, family structures, religion, education, and socioeconomic status among many more (4–21) . Most importantly, stigma has also been identified as one of the leading barriers in seeking mental health care treatment (19, 22), especially in the Arab World (23).

Within the Arab culture, there is a strong emphasis on the collective wellness of the group over that of the individual. For this reason, a strong attachment to social norms and values can be readily observed (5, 10). Because mental disorders are conceptualized adversely, stigmatization is considered very high in the Arab region (17, 18). This leads to perceived public mental illness stigma and self-stigma, causing a delay in active help-seeking behavior (10, 17, 18, 22). Saving face to avoid embarrassment, especially to protect family honor (5, 24) and out of fear of being labeled by others as “crazy” or “majnoun,” is common (18, 24).

Despite growing challenges, efforts have been made to address this wide treatment gap, although resources remain scarce and national legislation supporting the beneficiaries of treatment is insufficient (1–3, 5) . There has also been a call to conduct more clinical research in the Arab region, investigating conceptualizations of mental illness, available treatment options, help-seeking behaviors, quality of mental health services, patient satisfaction, and perceptions on mental health (9, 15, 17, 18). The need for further research to guide appropriate treatment services for the development of effective and efficient psychiatric care in Jordan is evident. There also seems to be a call for primary mental health care clinicians to assist in removing already existing barriers and attempting to reduce biases toward psychotherapy by looking more deeply into the therapeutic encounter (14, 25,). Therefore, a better understanding of the currently existing mental health care structures and perspectives on existing psychological services is urgently needed. The present study is the first to our knowledge that investigates the perceptions, preferences, and expectations of both members of the host and Syrian refugee community regarding psychotherapy in Jordan. Moreover, the study allows clinicians and researchers to gain preliminary insight into the current practices of mental health services in Jordan from the perspective of mental health care beneficiaries. Patient expectations, preferences, and perceptions pertaining to the local mental health care providers were collected and covered topics that include patient satisfaction with services, biases toward therapy, expectations from therapists, perceptions of the therapeutic relationship, effects of therapy, and finally stigma.

## Methods

### Participants

In total, a sample of 100 patients receiving psychotherapeutic expert interventions were recruited between October and December 2017 in Amman, Jordan. Paper-and-pencil interviewing (PAPI) took place, whereby a trained international or local interviewer read the question to the participants and filled out the answers into the questionnaire in Arabic. Before the beginning of data collection, all interviewers received in-depth training to ensure consistency in the interview process.

Inclusion criteria for the current research study were defined as the following: 1) individuals aged between 18 and 75 years; 2) belonging either to the Jordanian host or Syrian refugee community; 3) are currently or have been receiving psychotherapy or counseling sessions at organizations, which provide mental health services; 4) have attended a minimum of five sessions before the initial interview; 5) additional psychotropic treatment was allowed.

### Procedures

Grounded in this selection process, recruitment of a randomized sample was not viable. However, within the current study design, a balance between different patient groups with regard to sociodemographic variables, such as gender and background (host and Syrian refugee community), was established.

As a sampling method, cooperating local organizations contacted possible study participants directly and asked for their willingness in participating. Next, suitable patients who gave their consent were contacted *via* telephone by local staff of Jiyan Foundation and CH4S. Recruitment was conducted throughout the following mental health service providers in Jordan: Al Hashmi Clinic, Bright Future, Caritas Jordan, CharitéHelp4Syria, Center for Victims of Torture, Institute for Family Health, Nippon International Cooperation for Community Development, Happiness Again, Saudi Hospital, and Médicins du Monde. A signed informed consent was requested from all patients who met the inclusion criteria before officially joining the study.

Because of logistic challenges, a matching of gender between interviewers and participants could not always be ensured. All participants were reimbursed for their travel costs. Furthermore, all respondents received a CH4S telephone hotline number in case follow-up psychological support was needed. After completion of the pencil-and-paper questionnaires, data were translated into the English language by local translators and entered into a spreadsheet using the Statistical Package for the Social Sciences (SPSS) 25, iOS. The study design has been approved by the ethical committees of Charité-Universitätsmedizin, Berlin, Germany, the Department of Health (DoH), and the Ministry of Health (MoH), Amman, Jordan.

### Questionnaire

Because of the scarcity of appropriate and culturally sensitive questionnaires, which measure patient perceptions and preferences regarding psychotherapy in the Arab world, a comprehensive assessment tool was developed in close collaboration with the NGO Misereor for this research project. The Patient Satisfaction Questionnaire (PSQ) was designed with an aim to assess patient perspectives on the process of psychotherapy, including beliefs, perceptions, and expectations regarding patient satisfaction, perceived bias, stigma, and the effects of therapy in Arabic-speaking countries.

During the development of the questionnaire, regular feedback concerning the acceptability, feasibility, and comprehensibility was collected through pilot trials, enabling continuous optimization until saturation was reached.

The PSQ is a 26-item brief self-report assessment tool consisting of four subscales, which cover the core domains of mental health care delivery: (1) therapeutic relationship, (2) bias, (3) effects of therapy, and (4) stigmatization (see details in **Tables 2** **–** **5**). Within the first domain, titled the therapeutic relationship (nine items), answers were scored on a 5-point Likert scale ranging from “very poor” (1), over “fair” (3), to “very good” (5). Concerning the second to the fourth domains, bias (six items), effects of therapy (seven items), and stigmatization (four items), all items were scored on a 5-point Likert scale, ranging from “totally disagree” (1), over “neither agree nor disagree” (3), to “totally agree” (5). In the present study, the four subscales displayed questionable and inconsistent to excellent consistency measures with Cronbach’s alpha ranging from 0.64 and 0.67 to 0.91 and 0.92, respectively (27).

### Statistical Analysis

All analyses were composed of descriptive and inferential statistics for the PSQ. Data were collected and stored electronically in a spreadsheet for a spreadsheet using the Statistical Package for the Social Sciences (SPSS) 25, MacOS-X. The central tendency of continuous measures was represented using frequencies, percentages, means, and the variability with standard deviations as well as the range of each variable. All categorical variables were represented with percentages along with the actual counts so that missing measures are apparent in **Tables 1** to **5**. To detect possible differences between host and refugee communities subsample analyses, it is indented to use either independent t-tests or a nonparametric test, the Mann-Whitney U test.

**Table 1:** Sociodemographic characteristics of the survey sample.

Sociodemographic data	*N* = 100
**Gender (%)**	
Male	39.0
Female	61.0
**Age (years, %)**	38.24 (13.98)*
18–28	31.0
29–39	30.0
40–54	24.0
55–74	15.0
**Ethnicity (%)**	
Arab	98.0
Mandaean	1.0
Other	1.0
**Religion (%)**	
Muslim	98.0
Christian	1.0
Mandaean	1.0
**Target Group (%)**	
Host community	35.0
Syrian refugee community	65.0
**Waiting time (%)**	
Same day	31.0
1–7 days	47.0
1–2 weeks	10.0
3–4 weeks	7.0
More than 4 weeks	4.0
Missing	1.0

Waiting time = Waiting time between registration with the organization and first contact with a physician or psychologist. *Mean and standard deviation for age.

**Table 2:** Descriptive Statistics of the Patient Satisfaction Questionnaire.

How was the doctor or nurse at…	Very poor (%)	Poor (%)	Fair (%)	Good (%)	Very good (%)	Mean (SD)
Making you feel at ease? (n = 99)	2.0	0	5.1	19.2	73.7	4.63 (0.76)
Letting you tell your story? (n = 99)	0	0	4.0	15.2	80.8	4.77 (0.51)
Really listening? (n = 98)	0	0	5.1	13.3	81.6	4.77 (0.53)
Being interested in you as a whole person? (n = 99)	0	0	5.1	13.1	81.8	4.77 (0.53)
Fully understanding your concerns? (n = 99)	0	1.0	11.1	17.2	70.2	4.58 (0.73)
Showing care and compassion? (n = 99)	0	1.0	4.0	21.2	73.7	4.68 (0.60)
Explaining things clearly? (n = 99)	0	1.0	3.0	10.1	85.9	4.81 (0.53)
Helping you to take control? (n = 99)	0	1.0	3.0	18.2	77.8	4.73 (0.57)
Overall rating of the consultation treatment (n = 99)	0	0	6.1	19.2	74.7	4.69 (0.58)
**Overall rating on the Patient Satisfaction Questionnaire**	**0.2**	**0.4**	**5.2**	**16.3**	**77.8**	**4.71 (0.59)**

The full sample consisted of N = 100 participants. Very poor = 1, Poor = 2, Fair = 3, Good = 4, Very good = 5.

**Table 3:** Descriptive statistics of the Bias Questionnaire.

It is acceptable if the therapist…	Totally disagree (%)	Somewhat disagree (%)	Undecided (%)	Somewhat agree (%)	Totally agree (%)	Mean (SD)
Is a man.	17.0	13.0	24.0	25.0	21.0	3.20 (1.37)
Is a woman. (n = 99)	2.0	4.0	14.1	22.2	57.6	4.29 (0.99)
Has a different opinion regarding national politics.	21.0	12.0	48.0	13.0	6.0	2.71 (1.12)
Is from another country.	1.0	2.0	29.0	22.0	46.0	4.10 (0.96)
Belongs to a different ethnic group. (n = 99)	8.1	2.0	35.4	32.3	22.2	3.59 (1.11)
Belongs to a different religious group.	13.0	10.0	31.0	22.0	24.0	3.34 (1.30)
**Overall rating on the Bias Questionnaire**	**10.4**	**7.2**	**30.3**	**22.7**	**29.4**	**3.54 (1.14)**

The full sample consisted of N = 100 participants. Totally disagree = 1, Somewhat disagree = 2, Neither agree nor disagree = 3, Somewhat agree = 4, Totally agree = 5.

**Table 4:** Descriptive statistics of the Effects of the Therapy Questionnaire.

	Totally disagree (%)	Somewhat disagree (%)	Undecided (%)	Somewhat agree (%)	Totally agree (%)	Mean (SD)
I mostly feel relieved after the therapy sessions.	0	4.0	4.0	18.0	74.0	4.62 (0.75)
The therapy helped me to handle my problems and my distress.	1.0	2.0	6.0	34.0	57.0	4.44 (0.78)
Now I can understand much better where my problems came from.	1.0	3.0	8.0	25.0	63.0	4.46 (0.85)
With therapy, it is easier for me to face the difficulties in my life.	1.0	2.0	9.0	35.0	53.0	4.37 (0.81)
Therapy gave me new hope and new perspectives for my life.	1.0	2.0	10.0	27.0	60.0	4.43 (0.83)
I now get along better with the people in my immediate environment.	1.0	2.0	9.0	28.0	60.0	4.44 (0.82)
Therapy helped me to find solutions for my problems.	1.0	1.0	10.0	35.0	53.0	4.38 (0.79)
**Overall rating on the Effects of the Therapy Questionnaire**	**0.8**	**2.3**	**8.0**	**28.8**	**60.0**	**4.44 (0.80)**

The full sample consisted of N = 100 participants. Totally disagree = 1, Somewhat disagree = 2, Neither agree nor disagree = 3, Somewhat agree = 4, Totally agree = 5.

**Table 5:** Descriptive statistics of the Stigma Questionnaire.

	Totally disagree (%)	Somewhat disagree (%)	Undecided (%)	Somewhat agree (%)	Totally agree (%)	Mean (SD)
I think if others know about my psychological problems, they lose respect for me.	39.0	25.0	5.0	20.0	11.0	2.39 (1.45)
I am afraid of possible disadvantages in regard to my family planning and family life because of my psychological problems. (n = 99)	26.3	21.2	1.0	26.3	25.3	3.03 (1.60)
I am scared that people are thinking or talking about me in a negative way because I am in therapy for my psychological problems.	34.0	20.0	7.0	27.0	12.0	2.63 (1.48)
I feel ashamed that I have to go to a therapist for my problems. (n = 99)	63.6	18.2	2.0	10.1	6.1	1.77 (1.25)
**Overall rating on the Stigma Questionnaire**	**40.7**	**21.1**	**3.7**	**20.9**	**13.6**	**2.45 (1.44)**

The full sample consisted of N = 100 participants. Totally disagree = 1, Somewhat disagree = 2, Neither agree nor disagree = 3, Somewhat agree = 4, Totally agree = 5.

**Table 6:** Descriptive statistics and analysis of subsample differences for all subscales.

Outcome Variable	Mean (SD)	*P*
Satisfaction Subscale		
Host community (*n* = 32)	4.72 (0.55)	0.26^a^
Refugee community (*n* = 65)	4.70 (0.40)	
Bias Subscale		
Host community (*n* = 33)	3.37 (0.67)	0.12^b^
Refugee community (*n* = 65)	3.61 (0.71)	
Effects of the Therapy Subscale		
Host community (*n* = 33)	4.48 (0.78)	0.27^a^
Refugee community (*n* = 65)	4.43(0.60)	
Stigma Subscale		
Host community (*n* = 33)	2.74 (1.18)	0.11
Refugee community (*n* = 65)	2.23 (0.89)	

^a^Mann-Whitney U Test (two-tailed); ^b^independent samples t-test; α = 0.05 (two-tailed)

## Results

For the current study, 100 participants, 39 males and 61 females, were recruited, ranging in age from 18 to 74 years. A vast majority of 98% stated Arab as their ethnicity and indicated Islam as their religious affiliation. Two-thirds of the sample were from the Syrian refugee community (65%) whereas one-third was of Jordan origin (35%). **Table 1** presents all assessed sociodemographic characteristics of the sample in detail.

Overall, descriptive results of frequencies concerning each of the four subscales are displayed in **Tables 2** to **5**. Additionally, subsample analysis between the host and Syrian refugee community members was performed. Mean scores of each subscale and results of each tests are displayed in **Table 6**. Lastly, depending on normal or nonnormal distribution of data, one independent t-test and three Mann-Whitney U tests were conducted, revealing no significant differences between all four subscales in both subsamples: *U*_Satisfaction_ = –1.13, *p* = 0.26, *t*_Bias_(96) = –1.576, *p* = 0.12, *U*_EffectsofTherapy_ = –1.07, *p* = 0.27 and *U*_Stigma_ = –1.62, *p* = 0.11. Therefore, the current results present total responses of participants.

### Patient Satisfaction

Participants rated their satisfaction regarding the consultation and treatment of the therapist on eight items. Overall, results showed high satisfaction rates on the subscale, with an average mean of 4.71 (0.59). In total, 77.8% rated the interpersonal level of treatment as “very good” and 16.3% as “good.” Only 0.2% described experienced treatment delivery as “very poor” and 0.4% as “poor,” whereas 5.2% rated it “fair.” **Table 2** illustrates all descriptive statistics of the patient satisfaction subscale on an item level as well as overall.

### Bias

Attitudes in terms of bias toward the therapists were rated on a subscale with six items. In total, bias against the therapist was relatively low; instead, the average response of 3.54 (1.14) indicates high rates of acceptability. With 29.4% stating “totally agree” and 22.7% “somewhat agree,” a majority of 52.1% accepted possible differences in gender, religious affiliation, ethnicity, or political attitudes with their therapist. Still, 10.4% expressed their disapproval with “totally disagree” and 7.2% with “somewhat disagree,” indicating minimal rates of bias against their therapist, whereas 30.3% remained “undecided.” **Table 3** depicts all descriptive statistics of the bias subscale for all six items and overall scores.

### Effects of Therapy

Concerning the effects of therapy, the overall mean of participants displays overall high rates of approval, with 4.44 (0.80SD) on average. In total, 60% of the respondents stated “totally agree,” whereas 28.8% endorsed the beneficial effects with “somewhat agree,” resulting in 88.8% of respondents who expressed positive effects of the therapy. Merely 3.1% believed that the therapy led to no favorable effects, with rates at 0.8% for “totally disagree” and 2.3% for “somewhat disagree,” respectively, whereas 8.0% were undecided. **Table 4** represents all descriptive statistics of the effects of therapy subscale regarding average scores and on the individual level of the seven items.

### Stigma

Because all four items on the stigma subscales were phrased with reversed items, lower rates of approval indicate lower degrees of stigma. A total mean score of 2.45 (1.44SD) demonstrates moderate rates of self-stigma. However, the mean value does not reflect respondents’ neutrality; instead, many participants either indicated self-stigma with 20.9% for “somewhat agree” and even 13.6% for “totally agree” or rejecting self-stigma with 40.7% stating “totally disagree” and 21.1% “somewhat disagree,” whereas only 3.7% responded “undecided.” Answer patterns across all four items and average scores for the stigma subscale are shown in **Table 5**.

## Discussion

To our knowledge, this is the first exploratory study that has been conducted in Jordan investigating both refugee and host community perceptions regarding patient satisfaction, bias, stigma, and the effects of psychotherapy. Main results showed i) a high level of satisfaction with the treatment, ii) a high level of acceptance toward the therapist, iii) an overall positive evaluation of the treatment process, and finally iv) a moderate level of self-stigmatization. Because knowledge and information are scarce regarding the attitudes and opinions of Arabs and refugees on the process of psychotherapy, this study offers the first insights from the perspective of beneficiaries who have had contact with mental health services in Jordan.

Understanding patient satisfaction and the effects of therapy are important indicators for health care quality (28). Perspectives of patients are being considered at an increasing rate in the development and improvement of mental health care services and treatment interventions (29). In the current study, scores for patient satisfaction and effects of therapy seem to be quite high, and the overall consultation and its benefits were rated mostly as “very good.” Our study participants reported feeling heard, understood, empathized with, and were able to take control over their issues. They also mentioned feeling relieved after sessions, understood their problems better, had hope and coped better. There are several possible explanations for the high scores concerning the patients’ satisfaction and effects of therapy. One reason could be that our study participants seemed to have experienced a short waiting period of 1 to 7 days. Short waiting periods have been shown in multiple trials to improve a persons’ therapy experience (29,).

As previously mentioned, there was a high level of overall satisfaction reported in our study. Furthermore, the majority of the participants in the current sample (61%) ranged between 18 and 39 years. An interesting study reported the highest level of satisfaction to be observed in the age group of 18 to 34 (24), whereas another study mentioned that older patients tend to rate higher on satisfaction scores (31). Therefore, it seems that available research on the relationship between age and reported level of satisfaction with treatment in this cultural group is scarce and contradictory. Furthermore, in another study, Ghuloum, Bener, and Burgut (28) also found that, compared to Arab expatriates, Spanish patients’ ratings of patient satisfaction with mental health care services were significantly lower than those in the Arab group. This gives light to the need for further studies with larger sample sizes and more rigorous research methodology to explore these discrepancies in the literature. High levels of satisfaction may also be a result of the characteristics of our participants of whom the majority are refugees who are in need of humanitarian aid assistance (32), unlike most cohorts in other studies with higher socioeconomic status and better mental health care. Although refugee populations are at great risk for developing psychopathology (33–35), there were no significant differences observed between host and refugee community members in our study. Perhaps the need for psychological support coupled with short waiting periods and a good therapeutic alliance may have had a positive impact on patient satisfaction and effects of therapy ratings. It would be of major interest in future research to see this hypothesis being investigated in more detail.

In 1975, Luborsky, Singer, and Luborsky investigated the hypothesis that regardless of the type and method of psychotherapy, across different treatments, only small differences in effect sizes were observed when comparing active treatments (36). Since then, several meta-analyses have confirmed the existence of this phenomenon, also known as the “Dodo Bird’s Verdict” (36–39). In a nutshell, it seems that certain “active ingredients” exist, which are shared among all psychotherapeutic models (e.g., feeling heard, being understood, validated, and accepted) and influence patient satisfaction in therapy (31–40).

Overall, there were no major biases toward psychotherapists observed in our study. In general, participants seemed to have no reservations regarding their therapists’ religious orientation, country of origin, and ethnic background. Only two biases appeared worthy of further investigation, one being gender related and the other concerning a difference in opinion about their national politics. Some research in this field hypothesizes that, within the Arab culture, there is a clear preference to and acceptability of females as therapists (24, 41). At this point, it is important to take into consideration our unique sample size, of which the majority are women. Regarding gender preferences in clinicians, women may feel more comfortable with a female therapist or medical doctor, so that they can address gender-related issues more openly and with greater ease (41). Furthermore, in the Arab world, studies have reported that, as a part of cultural norms, women are often assigned a caretaker role that is expected to nurture and care for others (41, 42). Therefore, it is not surprising that, in our research, both male and female participants seemed to find it more acceptable for a woman to be a therapist.

Regarding the bias of differences in political opinion between therapist and client, it seems like a general pattern emerged, ranging between undecided and disapproval. The ambivalence in their response may be because participants are mostly individuals who are fleeing from war and others from the host community who are impoverished (8, 9). Ongoing conflicts in the Middle East have led many of these individuals to seek psychiatric treatment. Nonetheless, these results should be interpreted cautiously.

Across the literature, various social barriers have been identified in the access and delivery of mental health services (5, 10, 17, 18). This may explain why there is more variability in patient responses concerning stigma. Several factors have been pointed out by researchers for increasing public stigma and bias toward mental health services (4). Some of these include gender and socioeconomic status (12). Further studies have also identified that being an immigrant and coming from a certain cultural group also increase public stigma (20). Other studies found Muslim women to be twice as likely compared to males to show positive attitudes toward help-seeking behaviors and thus be more open to seeking treatment (42). With regard to the specific items in our questionnaires, participants showed some ambivalence when asked whether others would lose respect for them if they knew about their psychological problems: 64% of the sample disagreed to this statement, whereas 31% agreed, and only a small number remained undecided. The items concerning fear of judgment and feeling ashamed were also ambivalently rated. Stigma has been known to be relatively high in the Arab world. In contrast, our exploratory study shows a different perspective. Although there is a scarcity in the availability of mental health care services, the need is being addressed, and public efforts for psychological awareness have also increased in recent years (1– 3, 5). This could explain a decrease in the currently recognized pattern within Arab cultures and refugee groups.

Finally, regarding the item on family planning, there seemed to be a clear split in the responses toward this item. Although almost half of the cohort (47.5%) disagreed to the possible disadvantages concerning family planning and family life because of psychological problems, the other half (51.5%) agreed to it posing as a barrier. Family reputation is an integral feature for choosing a marriage partner within the Arab culture (41). Among some participants, seeking help may still be viewed as shameful and could “tarnish the family’s reputation” (41, 42).

This exploratory study gives a detailed insight into the perception of psychotherapeutic services in a large cohort of host and refugee beneficiaries in Jordan. Although samples were collected from a diverse number of hospitals and organizations, a potential selection bias in this convenience sample may have occurred during recruitment because more compliant and more open respondents may have agreed to take part in the study. This may also have affected general positive responses throughout the questionnaire and overall positive ratings. This sample was not randomized; however, the study design attempted to create a balance between host and refugee community. Furthermore, several methodological limitations were also present in this research project, namely, that no data were available on psychiatric diagnosis, current psychopathology, or total length of treatment. It would also be ideal if future research could focus more on collecting data from qualitative interviews. This approach would allow for rich and in-depth further investigations that were not within the scope of this study.

In conclusion, although the preliminary results should be interpreted with caution, the present study provides evidence toward a positive attitude toward psychotherapists and psychotherapeutic treatment in both the refugee and host communities in Jordan. These findings validate the argument that, in an environment where psychiatric disorders are usually stigmatized, contact to mental health services not only improves individual well-being but also positively alters attitude, at least in those receiving professional support. This knowledge may not only be useful in the case of Jordan but also be applied in other Arab countries with comparable mental health care structures. Further studies should be conducted with a more rigorous population selection, sample size, intervention description, and possibly a mixed-methods approach.

## Data Availability

The datasets generated for this study are available on request to the corresponding author.

## Ethics Statement

The study was carried out in accordance with the recommendations of the Ministry of Health of the Hashemite Kingdom of Jordan with written informed consent from all subjects. All subjects gave written informed consent in accordance with the Declaration of Helsinki. The protocol was approved by the Ministry of Health in the Hashemite Kingdom of Jordan. The original ethics approval is available and can be submitted upon request.

## Author Contributions

CK contributed to the interpretation of data, writing (sections: Introduction and Discussion), and revision. KB contributed to the writing and data analysis (sections: Methods and Results). EH contributed to the conceptualization of study design, revision, and supervision. SS contributed to the data collection and conceptualization of study design and logistics. JS contributed to the data collection and conceptualization of study design. MB contributed to the conceptualization of idea, study design, revision, and supervision. All authors read and approved the final manuscript.

## Funding

The study was funded by the German Ministry of Economic Cooperation and Development within the Special Initiative “Psychosocial Support for Syrian and Iraqi Refugees and Internally Displaced People.”

## Conflict of Interest Statement

The authors declare that the research was conducted in the absence of any commercial or financial relationships that could be construed as a potential conflict of interest.
